# Bacterial N-Acyl Homoserine Lactone Priming Enhances Leaf-Rust Resistance in Winter Wheat and Some Genomic Regions Are Associated with Priming Efficiency

**DOI:** 10.3390/microorganisms12101936

**Published:** 2024-09-24

**Authors:** Behnaz Soleimani, Heike Lehnert, Adam Schikora, Andreas Stahl, Andrea Matros, Gwendolin Wehner

**Affiliations:** 1 Institute for Resistance Research and Stress Tolerance, Federal Research Centre for Cultivated Plants, Julius Kuehn Institute (JKI), Erwin-Baur-Str. 27, 06484 Quedlinburg, Germany; behnaz.soleimani@julius-kuehn.de (B.S.); andreas.stahl@julius-kuehn.de (A.S.); andrea.matros@compolytics.com (A.M.); 2 Institute for Biosafety in Plant Biotechnology, Federal Research Centre for Cultivated Plants, Julius Kuehn Institute (JKI), Erwin-Baur-Str. 27, 06484 Quedlinburg, Germany; heike.lehnert@julius-kuehn.de; 3 Institute for Epidemiology and Pathogen Diagnostics, Federal Research Centre for Cultivated Plants, Julius Kuehn Institute (JKI), Messeweg 11/12, 38104 Braunschweig, Germany; adam.schikora@julius-kuehn.de

**Keywords:** *Puccinia triticina*, *Ensifer meliloti*, microbial priming, N-acyl homoserine lactone (AHL), microbiome, genome-wide association study

## Abstract

Leaf rust (*Puccinia triticina*) is a common disease that causes significant yield losses in wheat. The most frequently used methods to control leaf rust are the application of fungicides and the cultivation of resistant genotypes. However, high genetic diversity and associated adaptability of pathogen populations hamper achieving durable resistance in wheat. Emerging alternatives, such as microbial priming, may represent an effective measure to stimulate plant defense mechanisms and could serve as a means of controlling a broad range of pathogens. In this study, 175 wheat genotypes were inoculated with two bacterial strains: *Ensifer meliloti* strain *expR^+^ch* (producing N-acyl homoserine lactone (AHL)) or transformed *E. meliloti* carrying the lactonase gene *attM* (control). In total, 21 genotypes indicated higher resistance upon bacterial AHL priming. Subsequently, the phenotypic data of 175 genotypes combined with 9917 single-nucleotide polymorphisms (SNPs) in a genome-wide association study to identify quantitative trait loci (QTLs) and associated markers for relative infection under *attM* and *expR^+^ch* conditions and priming efficiency using the *Genome Association and Prediction Integrated Tool* (GAPIT). In total, 15 QTLs for relative infection under both conditions and priming efficiency were identified on chromosomes 1A, 1B, 2A, 3A, 3B, 3D, 6A, and 6B, which may represent targets for wheat breeding for priming and leaf-rust resistance.

## 1. Introduction

Wheat is the most important staple food for 35% of the world’s population [1], but its production is reduced by abiotic and biotic stressors. Leaf rust as a foliar disease caused by the biotrophic fungus *Puccinia triticina* significantly reduces yield by decreasing grain number per head and grain weight in large geographical areas [2,3]. However, the severity of leaf-rust damage depends on the wheat growing stage, e.g., grain yield was decreased more than 50% by the infection of plants at an early growth stage [4].

The cultivation of resistant varieties is the most effective, economic, and ecological method to reduce the negative impacts of *P. triticina* infection on grain yield. To date, 80 leaf-rust resistance genes are known in wheat. Most of them confer race-specific resistance [3]. The race-specific resistance or vertical resistance is controlled by a single gene and causes resistance to particular strains of pathogens. Therefore, this type of resistance is unstable. The effectiveness of race-specific resistances can be lost within a few years through the occurrence of new virulent pathogen strains [5]. Non-race-specific or horizontal resistance is controlled by many genes and provides more stable resistance that is not easily overcome by strains of the pathogen. Only 3 out of 80 identified resistance genes, i.e., *Lr34*, *Lr46*, and *Lr67*, were reported as non-race-specific [6].

The usage of beneficial plant–microbe interactions could be an alternative approach to protect plants against pathogens by inducing resistance to abiotic and biotic stressors. For instance, the positive effect of rhizobacteria in plant protection by induced systemic resistance (ISR) against pathogens has been reported in previous studies [7,8,9,10].

In nature, bacteria use concentration gradients of small molecules as chemical signals for their communication, i.e., producing, releasing, detecting, and responding [11]. Such molecules are referred to as quorum sensing (QS) molecules, which can be synthesized by Gram-positive and Gram-negative bacteria. The Gram-negative rhizobacteria often use N-acyl homoserine lactones (AHLs) as QS molecules in order to control their population behavior [12,13]. The AHL molecule is known as a priming inducer for the induction of systemic resistance in *Medicago truncatula*, *Solanum lycopersicum,* and other crops [9,14,15,16]. The acyl chain length of AHL molecules varies between 4 and 18 carbon atoms and is clustered into short-chained and long-chained AHLs. Plants react differently to specific AHL molecules according to the different acyl chain lengths of the AHL molecules. For instance, AHLs with six or eight carbon atoms promote plant growth in *Arabidopsis thaliana* [17]. Hernández-Reyes and Schenk [7] demonstrated that AHLs with 12 or 14 carbon atoms (oxo-C12-homoserine lactone (HSL) or oxo-C14-HSL) induced systemic resistance in several crop plants. The positive effect of oxo-C14-HSL against *Puccinia hordei* was reported in barley by Shrestha et al. [18], Wehner et al. [10], and Matros et al. [19]. Several studies are known that use seed priming to improve seed germination, plant development and productivity [20], drought stress tolerance [21], salinity tolerance, or fusarium head blight resistance [22] in wheat. To the best of our knowledge, no studies have examined the genotypic differences in priming efficiency (difference between relative infection under *attM* and *expR^+^ch* in the response to *P. triticina* infection ([10])) in wheat. Additionally, there is a lack of research on the impact of microbial seed priming on leaf-rust resistance and the identification of genomic regions associated with priming efficiency using genome-wide association studies (GWASs). The identification of genomic regions or candidate genes associated with priming efficiency could provide new resources to enhance leaf-rust resistance through bacterial AHL priming in wheat. The development of high-throughput sequencing technologies, such as next-generation sequencing or array-based technologies, enables us to generate comprehensive genotype data for entire plant genomes in a short time and with high accuracy [23] and to identify marker–trait associations and quantitative trait loci (QTLs) through mapping studies or GWASs. Genome-wide association studies were successfully used to identify genetic loci associated with traits of interest in different crops. Up to now, several studies have identified genetic loci associated with leaf-rust resistance on all wheat chromosomes [3,6,24,25].

In the present study, we aimed to establish a bacterial AHL-priming method in wheat and to achieve information about the genotype-specific priming effects on the response to leaf rust. Therefore, a diverse set of 175 wheat genotypes were treated with either *Ensifer meliloti* (*Sinorhizobium meliloti*) strain *expR^+^ch* producing AHL or transformed *E. meliloti* carrying the lactonase gene *attM* (non-AHL-producing, control) and then inoculated with leaf rust. Phenotypic and genotypic data (15K and 90K) were used to conduct GWASs in order to identify genome regions associated with the priming efficiency of wheat.

## 2. Materials and Methods

### 2.1. Plant Material

A diverse set of 175 winter wheat genotypes (Table S1) was selected out of 890 genotypes by using the k-medoids [26] clustering method [27] based on a modified Roger’s distance matrix [28]. The genotype set comprised 2, 25, 4, 101, 34, and 9 genotypes from Africa, Asia, Australia, Europe, USA, and of unknown origin, respectively. Wheat genotypes were analyzed by the 15K (25) or the 90K [29] iSelect chip (Table S1). The seeds and genomic data were kindly provided by Dr Dragan Perovic.

### 2.2. Priming and Phenotyping

Resistance of all 175 wheat genotypes was tested against leaf rust in the greenhouse at the Julius Kühn Institute, Quedlinburg, Germany. The greenhouse experiments were carried out using a split plot design. In all experiments, two genotypes, namely Tabasco and Borenos, were used as resistant and susceptible control genotypes, respectively. The control genotypes were used to evaluate the success rate of artificially infecting plants with *P. triticina.* All genotypes were tested in two independent experiments in 2020 and 2021, with three replications per genotype and three plants per pot (18 plants per genotype: two time points × three replications × three plants per pot). Each experiment was carried out in a greenhouse cabin with both *attM* and *expR^+^ch* treatment (Figure S1). The pots were placed on plates with 24 pots each (Figure S1). Seeds were germinated on wet filter paper in Petri dishes in the dark at room temperature. Seedlings were transplanted into pots with a volume (V = 7 cm × 7 cm × 6.5 cm) of 0.2 L of soil substrate (Fruhstorfer type T from HAWITA Gruppe GmbH) after 48 h. Cultivation conditions were 22 °C/18 °C (day/night temperatures) with 10 h of additional lighting and 50 to 55% humidity. The pots were irrigated with tap water (until their maximum holding capacity) and the substrate was maintained at consistent moisture for all accessions and treatments during the course of the experiment.

For bacterial AHL-priming induction, the soil substrate was treated with a bacterial solution of AHL-producing *E. meliloti* strain *expR^+^ch* or *E. meliloti* carrying the lactonase gene *attM* (non-AHL-producing bacterial control). The bacterial solution was prepared by cultivating bacteria for 48 h at 28 °C in 250 mL of tryptone–yeast medium with 10 mM CaCl_2_ containing 625 µL streptomycin (100 mg/mL) for *attM* and containing 625 µL streptomycin (100 mg/mL) and 250 µL kanamycin (100 mg/mL) for *expR^+^ch*. The bacterial solution was then centrifuged, and the bacterial pellets were resuspended in 10 mM MgCl_2_ and immediately used for inoculation. The soil substrate was treated with 4 mL of bacterial solution (OD_600_nm of 0.1, corresponding to 108 CFU/mL) each at 2, 8, and 14 days after planting (Wehner et al. 2019 [10]) using a multi-dispenser pipette equipped with a 50 mL tip vessel.

The wheat seedlings (16 days after planting, at BBCH 13 ([30]) were inoculated with *P. triticina* isolate ‘77WxR’ at the three-leaf stage. The spores *of P. triticina* were multiplied on susceptible wheat genotype ‘Borenos’ in the greenhouse and frozen at −80 °C immediately after harvest. The frozen spores were placed in a water bath (at 42 °C) for 10 min before use. Afterwards, twenty-five milligrams of spores of *P. triticina* (mixed with white clay 1:3) were used for the infection of 24 pots after spraying 0.01% Tween 20 solution on the leaves. The pot trays were covered with foil and incubated in darkness in containers (metal boxes in which the plants were placed and then covered with black plastic) for an additional 24 h.

Twelve days after artificial infection, the leaves were assessed for the percentage of diseased leaf area (*P. triticina* %, as a quantitative trait), which ranged from 1% to 80% on a scale where 100% is the maximum. Additionally, the infection type (*P. triticina* scores) was evaluated as a qualitative trait, ranging from 0 to 4 on a scale where 9 is the maximum. Relative infection (RI) was then calculated using the formula from Wehner et al. [10].

Relative infection (RI) = 0.2 × ln(*P. triticina* %) + (*P. triticina* scores)

Finally, priming efficiency (PE) was calculated for each genotype, as follows:

Priming efficiency (PE) = RI *attM* − RI *expR^+^ch*

### 2.3. Statistical Analysis

The statistics package SAS (2019, SAS Institute Inc., Cary, NC, USA) was used to analyze phenotypic data. The quality of phenotypic data was tested, and outliers were excluded. Specifically, trait values that were three times lower or higher than the standard deviation were defined as outliers and excluded from further analyses. The procedure PROC MIXED was used for the analysis of variances (ANOVA) and estimation of least square means (lsmeans). Lsmeans were used for the calculation of RI. In the following mixed model, μ is the general mean, α_i_ is the fixed effect of the ith priming effect, β_j_ is the fixed effect of the jth genotype, and α_i_ × β_j_ is the fixed interaction effect between the ith priming effect and the jth genotype effect. R_k_, E_l_, and P_m_ were considered random effect of kth replication, lth experiment, and mth plate, respectively. The e is the random error term.

Y = µ + α_i_ + β_j_ + α_i_ × β_j_ + R_k_ + E_l_ + P_m_ + e_ijklm_

Pearson correlation coefficients were estimated to compare the results of different experiments.

Finally, the repeatability (r^2^) was calculated between replications according to the following formula:

r^2^ = V_β_/(V_β_ +  V_βE_/E  +  V_e_/RE)

where E is the experiment, R is the replication, and V_e_ is the total error variance.

### 2.4. Genotypic Data and Genome-Wide Association Study (GWAS)

Two sets of 122 and 53 wheat genotypes were genotyped by 15K [26] or 90K [29] iSelect chip, respectively (Table S1). Genotyping was performed at SGS (Traitgenetics Section, Gatersleben, Germany). Common markers between the two genotyping platforms were identified based on their identifier (marker name), and identical markers between the two platforms were mapped on the reference genome of Chinese Spring RefSeq v2.1 based on their physical position [31]. A set of 10,541 SNPs were uniquely mapped on the reference genome. This marker set was filtered for markers with more than 30% missing values. The filtered SNP markers were imputed with standard parameters using the Beagle 4.1 software [32]. The imputed SNP markers were further filtered for minor allele frequency (MAF) > 5% and heterozygosity ≤ 12.5%. Finally, a set of 9917 SNP markers was retained and used for the identification of informative markers and GWASs. Informative markers were obtained according to their polymorphism information content (PIC) value and the map position of each marker.

To determine population structure and relatedness between genotypes, a set of 2567 informative markers was used. This marker set was selected using indep-pairwise 50 5 0.2 by PLINK v.1.07 (LD prune) [33], which comprises independent markers in linkage disequilibrium (LD).

Bayesian cluster analysis implemented in the structure [34] and principal coordinate analysis implemented in the DARwin 6 software [35] were used to determine population structure (methods are described in more detail in Soleimani et al. [26]). The Q matrix was selected based on the highest likelihood according to the result of the structure. An identical by-state matrix was calculated in R and was used as a kinship matrix (K-matrix).

To identify associations between genotypic and phenotypic data, a GWAS was conducted by using a Mixed Linear Model (MLM) in TASSEL (Trait Analysis by aSSociation, Evolution and Linkage [36]), a Compressed Mixed Linear Model (CMLM) in GAPIT (Genome Association and Prediction Integrated Tool ([37]), and FARMCPU (Fixed and random model Circulating Probability Unification) in GAPIT [38].

The MLM and CMLM comprised K-matrix and Q-matrix as corrections for population structure and relatedness. The Q-matrix was applied as a correction for population structure in FARMCPU. The *p*-values of marker–trait associations were adjusted by Bonferroni–Holm (−log_10_ (*p*-value)  ≥  5.3) correction. Markers with −log_10_ (*p*-value) ≥  5.3 could be found under both *attM* and *expR^+^ch*. No significant marker was identified at −log_10_ (*p*-value)  ≥  5.3, which was uniquely identified under *attM* and *expR^+^ch*. Therefore, markers with −log_10_ (*p*-value)  ≥  3 were considered significant.

All significantly associated markers (at −log_10_ (*p*-value)  ≥  3) were assigned to QTL regions based on their chromosomal position and the estimated LD decay (3.3 million base pairs). The LD decay was estimated as squared allelic correlation (r^2^) between all pairs of markers within a chromosome by using the software package R [39], therein the packages “genetics” and “LDheatmap” [40,41]. The genetic distances between markers in base pairs were plotted against the estimated r^2^. The r^2^ values were set to 0.2, and a locally weighted polynomial regression (LOESS) curve was fitted. Finally, the intersection of the LOESS curve and the critical r^2^ value were used to determine the LD decay [42,43]. The marker with the highest −log_10_ (*p*-value) value per QTL region was defined as the peak marker of this QTL region.

The identified QTL regions were compared with reported QTLs known for leaf-rust resistance from previous studies. All identified flanking sequences of associated markers for leaf rust were mapped to the reference genome sequence of Chinese Spring, allowing the assignment of candidate genes according to published functional gene annotations of the reference genome of Chinese Spring RefSeq v2.1 [31]. Several high-confidence (HC) genes were identified within all QTL regions. The marker position of significantly associated markers and the position of HC genes were compared. HC genes directly associated with a significantly associated marker or the HC gene, which is the nearest to a significant marker, were defined as the most interesting candidate genes. These candidate genes were discussed in more detail.

## 3. Results

### 3.1. Phenotypic Data

Two and three percent of the data for *expR^+^ch* and *attM* were identified as outliers, respectively, and subsequently excluded from further analysis. Decreased mean values for the percentage of leaf area diseased (in %) and infection type (score) after *expR^+^ch* compared to *attM* inoculations reflected the reduced RI values (Figure S2). These results indicated reduced susceptibility induced by bacterial AHL priming (Table 1).

The high and positive correlation (Table S2) was found between experiments for *Puccinia triticina* (scores) under *attM* (r ≥ 0.87) and *expR^+^ch* (r ≥ 0.88). A moderate correlation for *Puccinia triticina* (%) was observed between experiments under *attM* (0.51 < r < 0.54) and *expR^+^ch* (0.52 < r < 0.58).

The ANOVA analysis for RI indicated significant (*p* < 0.001) effects of genotype, priming effects, and interaction between genotype and priming effects (Table 2).

**Figure 1:**
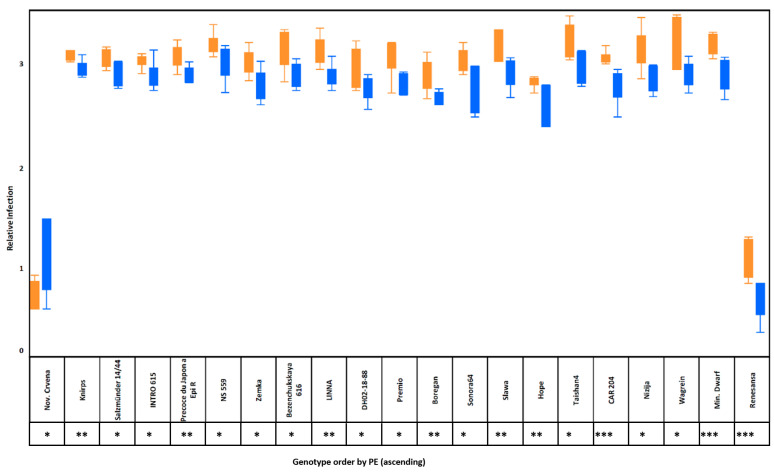
Whisker box plot of relative infection for twenty-one genotypes, which indicates a significant difference between *attM* (orange) and *expR^+^ch* (blue) treated plants. Significant difference between treatments for each genotype is shown with the star (*: *p* < 0.05, **: *p* < 0.01 and ***: *p* < 0.001, respectively).

In our study, 151 out of 175 genotypes (86.29%) showed a positive bacterial AHL-priming efficiency, which was indicated by reduced relative infection (Tables S1 and S3). However, only 21 genotypes showed significant (*p* < 0.05) differences in relative infection rate when compared between *attM* and *expR^+^ch* (Figure 1) treatments. One genotype (Gtyp0791, cv. Nov. Crvena) showed an increased relative leaf-rust infection rate upon bacterial AHL priming. These results suggested that the strength of response to AHL-producing bacteria is genotype-dependent.

### 3.2. Genotypic Data

**Figure 2:**
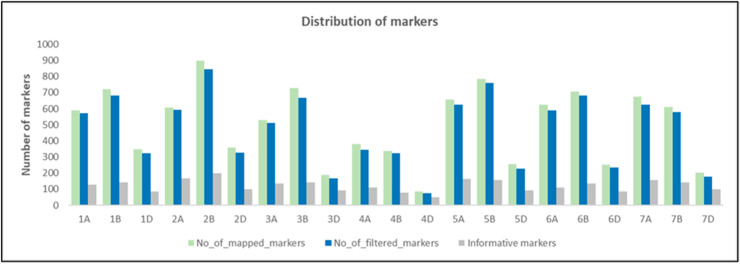
Distribution of markers across chromosomes. Colored bars indicate the number of mapped markers on the reference genome (green); filtered markers representing missing value, MAF, and heterozygosity (blue); and informative markers (grey), respectively.

Genotyping of the 175 genotypes by using the 15K and 90K iSelect chips resulted in a marker set of 12,908 and 81,587 SNPs, respectively. Out of this, 12,896 SNPs were identified as common markers that are included in both marker data sets. From those SNPs, a set of 10,541 markers could be uniquely mapped on the reference genome of Chinese Spring (RefSeq v2.1) based on physical positions (Figure 2). In total, 624 markers were excluded from further analyses due to missing values (30%), MAF (≥5%), and heterozygosity (≤12.5%). Finally, a set of 9917 SNP markers remained and was used to conduct the GWAS.

In total, 3858, 4532, and 1527 markers were found on the A, B, and D genome of wheat, respectively (Figure 2). The number of markers ranged between 76 SNPs (chromosome 4D) and 845 SNPs (chromosome 4B, Figure 2). Based on LD prune, 2567 SNPs out of 9917 markers were selected as informative markers, which were distributed across all wheat chromosomes. This set of markers was used to estimate the kinship matrix and determine population structure.

#### Population Structure

**Figure 3:**
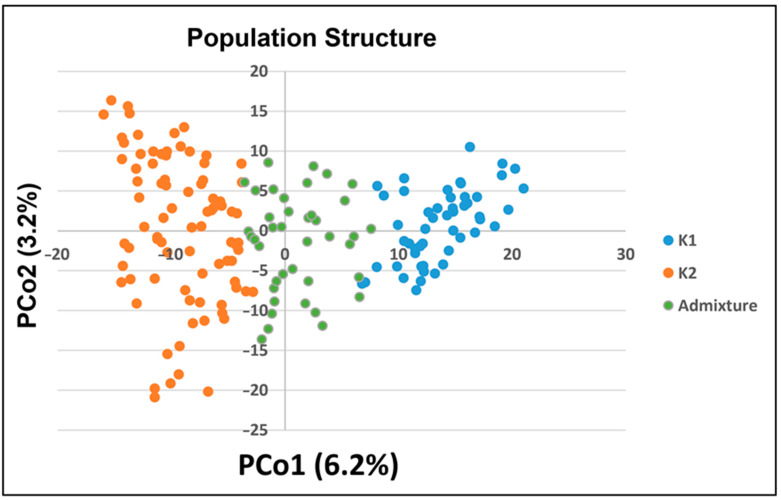
Principal coordinate analysis according to structure grouping of 175 wheat genotypes. Legend: blue = genotypes assigned to structure group 1, orange = genotypes assigned to structure group 2, and green = genotypes assigned to the admixed group.

The Bayesian clustering analysis in STRUCTURE revealed an optimal number of two subpopulation clusters (K = 2, Figure S3 and Figure 3). Genotypes were clustered according to their membership coefficients. Genotypes with a membership coefficient ≥ 0.7 to one of the clusters were assigned to the corresponding cluster. Genotypes with a membership coefficient < 0.7 to one of the groups were assigned to the admixed cluster. In total, 53, 82, and 40 genotypes were assigned to cluster 1 (blue) or 2 (orange) or the admixed (green) cluster (Figure 3). Cluster K1 comprised 82.7% genotypes originating from Europe, 3.8% from South America and Australia, 1.9% from North America, and 7.7% without information about country of origin. Cluster K2 contained 47.0%, 25.3%, 18.1%, 2.4%, 2.4%, and 1.2% genotypes from Europe, North America, Asia, South America, Australia, and Africa, respectively. No origin information about the country of origin was available for 3.6% of genotypes in cluster K2. The admixed cluster comprised 62.5% genotypes from Europe, 12.5% from Asia, 5% from South America, 12.5% from North America, 2.5% from Australia, and 5% without information about country of origin. A Principal Coordinate Analysis plot was used to visualize the results of the Bayesian cluster analysis (Figure 3). The first Principal coordinate (PCo) explained 6.2% of the variability, and the second PCo explained 3.2%. The results of both analyses indicated a weak to moderate population structure. No distinct clusters were observed based on origin or different plant breeding status.

The set of 9.917 SNPs was used to calculate LD (r^2^ ≥ 0.2) across and within wheat chromosomes. An LD value of 3,304,827 bp was obtained across all 21 wheat chromosomes (Table S4).

### 3.3. GWASs

Genome-wide association analyses were conducted to identify significant marker–trait associations for the three studied traits. To identify the most appropriate method for GWASs, three different models were tested, i.e., MLM in TASSEL, CMLM in GAPIT, and FARMCPU in GAPIT. Q- and K-matrix were used as corrections for population structure and relatedness. Based on optioned *p*-values, in all three tested methods, CMLM was identified as the best method to identify significant markers using GWASs (Figure S4).

**Figure 4:**
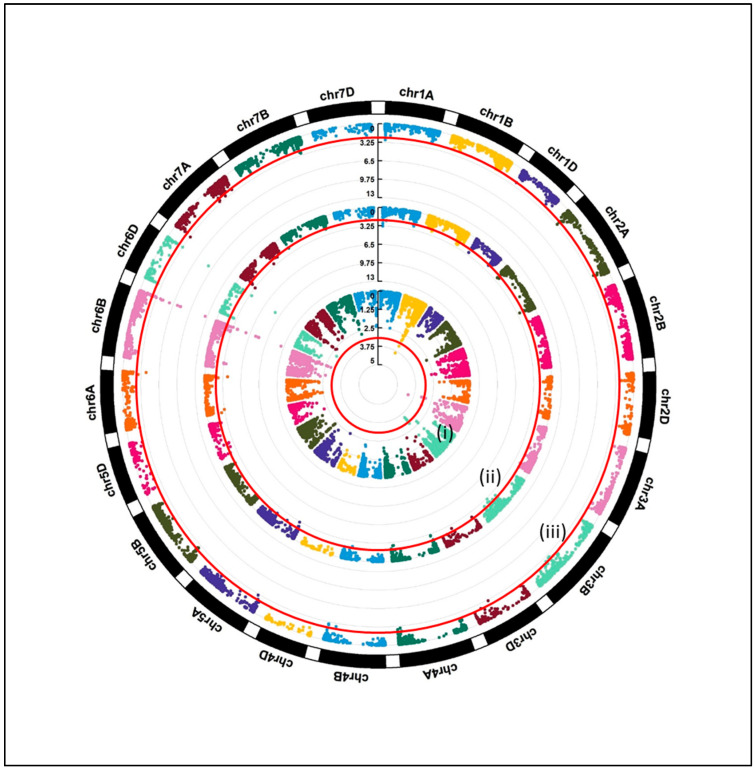
Circular Manhattan plot showing GWAS results for PE (**i**), RI for *expR^+^ch* (**ii**), and RI for *attM* (**iii**), respectively. From inner to outer circle: (**i**), (**ii**), and (**iii**).

In total, 50 SNPs were associated (*p* < 0.001) with the traits under investigation, i.e., RI under *attM* (17 SNPs) and *expR^+^ch* (24 SNPs) and bacterial AHL-priming efficiency (9 SNPs, Figure 4). Thirteen out of the forty-one identified markers for RI under *attM* and *expR^+^ch* (17 and 24 SNPs for *attM* and *expR^+^ch*, respectively, (Table S5)) were not considered to assign QTL regions and therefore excluded from further analysis. We did not consider common markers for assigning in the QTL region. The remaining markers, a set of 24 SNPs, were uniquely identified for either *attM*, *expR^+^ch,* or priming efficiency, and they clustered into 15 QTLs (Table 3).

For RI under *attM* conditions, four significant SNPs were identified and assigned to three QTLs on chromosomes 2A, 3D, and 6B (Table 3). The phenotypic variation varied between 30% (for identified markers on chromosome 2A and 3D) and 31% (RAC875_c56205_127 on chromosome 6B), respectively. GWASs identified eleven significantly associated markers for RI after *expR^+^ch*. These markers were located on the four wheat chromosomes 1A, 3B, 6A, and 6B and clustered into seven QTLs. The highest number of associated markers was found on chromosome 6B for this trait.

The 15 identified QTL regions were further analyzed to identify high-confidence (HC) genes within QTL regions. In total, 1224 annotated genes (Table S6) according to the Chinese Spring reference genome (RefSeq v2.1) were identified in these QTL regions. The nearest candidate gene to each identified SNP marker was selected as a potential candidate gene. Twenty-one genes were identified on chromosomes 1A, 1B, 2A, 3A, 3B, 3D, 6A, and 6B, which are described to be involved in biotic and abiotic stress responses (Table 3 and Table S7).

Eleven and six out of twenty-one identified candidate genes were associated with RI under the *expR^+^ch* condition and PE, respectively. Four out of eleven identified genes on chromosomes 6A and 6B were considered important genes due to their role in plant response to different environmental stressors. These genes are cellulose synthase, NBS-LRR-like resistance protein, protein kinase, and callose synthase. In addition, on chromosomes 3A and 3B, two genes encoding for a peroxidase and the serine/threonine-protein kinase ATM were associated with one and four markers for PE, respectively. These genes may be involved in plant responses to pathogen attack.

## 4. Discussion

Interaction between root and bacterial signal molecules, such as AHL, in the rhizosphere may lead to induced systemic resistance that could increase health, growth, and defense in plants [44]. This effect was used in different plants such as *Solanum lycopersicum*, *Arabidopsis thaliana*, *Glycine max,* and *Hordeum vulgare* to enhance resistance against different pathogens under greenhouse conditions [9,10,45,46]. In this study, the existing knowledge was transferred to wheat to obtain information about the genotype-specific bacterial AHL-priming effect on the response to leaf rust. The experiments followed methods that were already reported by Wehner et al. [10] for barley. The high and positive correlation between experiments indicated that experiments are comparable and reliable (Table S2). The beneficial role of AHL molecules and their ability to induce systemic resistance in plants was proven by previous studies [9,14,15,16,47] and could be observed in the current study as well. By bacterial AHL priming, the RI was reduced, which enhances PE using the wheat-*P. triticina* pathosystem. However, genotypic differences in priming efficiency were observed. To get more information about the genotype-specific priming response, genotypes were grouped according to their origin or breeding status. However, there was no connection between good priming efficiency and the origin or the breeding status of genotypes, respectively (Figure 3). These findings indicate that good priming efficiency is not dependent on the geographical origin of a genotype and that breeding activities in the last decades have had no influence on priming efficiency, as genetic resources or old cultivars of wheat, respectively, showed no differences in priming efficiency compared to modern wheat cultivars. As concluded from our previous studies on barley [10], AHL reduces susceptibility in a species- and genotype-specific manner. In the present study, 151 out of 175 genotypes showed a positive PE. Twenty of these genotypes indicated significant (*p* < 0.05) differences between *attM* and *expR^+^ch* treatments, and these genotypes were defined as primable genotypes. Notably, one genotype, namely “Nov. Crvena”, showed significant negative PE (*p* < 0.05) after *expR^+^ch* treatment. To address the negative and positive effects of bacterial AHL priming, further analyses are needed to better understand priming mechanisms related to plant resistance to different pathogens. In this regard, no evidence indicating the positive or negative effects of priming with *E. meliloti* on wheat seedlings has yet been reported. In barley, Shrestha et al. [18] and Wehner et al. [10] reported variable interaction between barley and *E. meliloti*, which reduced *P. hordei* infection in 44% of the tested barley genotypes. A varying response between barley cultivars was also observed for the AHL-mediated increased resistance to aphids [48]. In wheat, the general dependence of microbial priming-induced plant responses on the composition of the soil microbiome, as well as on the genotype, and vice versa, was reported [49]. More specifically, Salem et al. [50] reported a genotype-dependent growth response of winter wheat to inoculation with 1-aminocyclopropane-1-carboxylic acid-deaminase-containing bacteria under drought stress. In addition, recently, several *Rhizobium leguminosarum symbiovar viciae* strains were reported to be naturally competent to endophytically colonize wheat roots and induce root growth in a bacterial strain and wheat genotype-dependent manner [51].

The selection of the best suitable model for GWASs is required to reduce false-positive and false-negative marker–trait associations. The MLMs, including the Q- and K-matrix as corrections for population structure and relatedness, can reduce false-positive associations [38]. In our study, the most reliable results were achieved by CMLM compared to MLM and FARMCPU, according to a QQ plot based on *p*-values (Figure S4). In the present study, twelve significant associated markers for relative infection were identified (−log_10_ of the *p*-value ≥ 5.3) under *attM* and *expR^+^ch*. The twelve identified markers were excluded from further analysis because they were identified as common markers between both treatments. In general, several factors affect the results of a GWAS, i.e., the number and origin of genotypes, the distribution of markers, and the association model that is used. Due to the relatively small number of markers and the highly quantitative nature of the trait, it cannot be ruled out that the power of GWASs was scarce and that only a subset of major QTLs was identified by GWASs. In this regard, it could be assumed that high-density genotyping (i.e., deep GBS sequencing) of the same set of genotypes would result in a more precise QTL detection.

Previous studies identified QTLs that were involved in leaf-rust resistance (3, 23, 24). We identified 15 QTLs for RI under control and primed conditions, as well as for PE on chromosomes 1A, 1B, 2A, 3A, 3B, 3D, 6A, and 6B (Table 3). Candidate genes for each QTL region were identified, and genes involved in physiological processes under the primed condition and PE are discussed.

The positive effect of priming on resistance to *Puccinia triticina* could be explained by reducing the percentage of infected leaf area and relative infection under *expR^+^ch*. The relative infection is estimated based on the percentage and scores of *Puccinia triticina*. However, the effect of priming on *Puccinia triticina* (scores) is small compared to *Puccinia triticina* (%). The improvement in *Puccinia triticina* (%) can reduce the negative effects of fungal disease, such as reducing chlorophyll content, photosynthesis, and the accumulation of organic matter in photosynthetic source organs [52], by reducing the area of plants infected by *Puccinia triticina*, which can reduce the negative effect of fungal disease on wheat yield. Therefore, the measured traits might be useful traits for breeding approaches to improve priming efficiency by breeding.

Most interesting QTL regions were identified for RI after *expR^+^ch* treatment, such as QTL_RI_ *expR^+^ch* _1, which was located at chromosome 1A (from 8,138,538 bp to 14,738,538 bp) and for which the chaperone protein *DnaJ* was identified as the nearest candidate gene to the identified marker “Tdurum_contig43943_56” at a physical position 11,438,538 bp. The protein *DnaJ* is involved in various physiological processes, such as hormone regulation, but it also plays a role in disease resistance. *DnaJ* belongs to a large group of heat shock proteins, which were classified into different groups according to their molecular weight, of which *Hsp40* refers to *DnaJ* [53]. This is widely studied in different species, while knowledge about the *DnaJ* role in wheat is rather limited [54]. Liu et al. [53,54] indicated a role of this protein in defense against wheat yellow mosaic virus (*WYMV*) in wheat. Guo et al. [53] identified 119 *DnaJ* proteins in wheat. Therefore, due to the various physiological roles reported for *DnaJ*, its potential role in leaf-rust resistance could be interesting for further investigations. In addition, genes encoding *HSPs* were differently expressed under various abiotic and biotic stresses [53]. For instance, the suppression of *TaHsp90.2* and *Hsp90.3* compromised the hypersensitive response resistance against stripe rust disease in wheat [55]. Furthermore, Duan et al. [56] described the *TaHSC70* response to stripe rust via a JA-dependent signal transduction pathway in wheat. A marker significantly associated with bacterial AHL-priming efficiency, “wsnp_Ex_c1538_2937905”, was located at the 219,753,903 bp position on chromosome 3A (from 216,453,903 bp to 223,053,903 bp). As a candidate gene, the plant peroxidase-encoding gene was annotated, encoding for heme-proteins that are involved in various physiological processes, including plant defense against pathogen infection through signaling pathways mediated by salicylic acid, jasmonic acid, and ethylene [57]. The relationship between peroxidase activity and leaf rust was already reported by Johnson and Cunningham [58]; those results indicated that a low infection rate correlated with higher peroxidase activity in inoculated wheat plants. Later, Johnson et al. [59] evaluated the function of two peroxidase isozymes (Hi and Low peroxidase) in the responses of wheat carrying the *Lr 10* gene and demonstrated the role of both Hi and Low peroxidase in the low infection type of leaf rust. Southerton and Deverall [60] reported that changes in peroxidase activities in wheat cultivars resulted in altered resistance to leaf rust-causing fungus. In addition, enhanced leaf-rust resistance in wheat lines carrying the *Lr 35* gene could be correlated with increased peroxidase and chitinase activities at the flag leaf stage [61]. Similarly, Caruso et al. [62] pointed to the antifungal activity of peroxidase in the case of *Botrytis cinerea*, *Fusarium culmorum,* and *Trichoderma viride* in wheat. Therefore, this gene is considered a potential candidate gene that could increase leaf-rust resistance in wheat.

The effective role of R genes against pathogens, especially towards obligate biotrophic pathogens such as rust fungi, has been well studied in plant defense systems. These genes encode proteins containing a nucleotide binding site domain and an extended domain of leucine-rich repeats, which are classified into two groups: those with an N-terminal coiled-coil or a Toll-interleukin receptor-type domain [63]. In the present study, a gene encoding for an NBS-LRR-like resistance protein was identified as a key candidate for RI upon *expR^+^ch* at the physical position 82,951,636 on chromosome 6B (from 82,951,636 bp to 82,951,636 bp). Despite intense research, this gene was not yet identified among leaf-rust resistance genes [64]. Notably, the region was also assigned among the consensus genomic regions conferring leaf-rust resistance in wheat in a recent meta-QTL analysis with the confidence interval 81.2–84.4 cM on chromosome 6B [65].

Protein kinases, as main regulatory components, are involved in different cellular functions [66], and they comprise a large protein family in plants. These proteins are clustered in different groups, of which mitogen-activated protein kinases (MAPKs) are the largest and most important. The MAPKs are involved in signaling during plant defense against pathogen attack [67] and abiotic stress [68]. In the present study, we identified four different genes encoding for kinases on chromosome 3B (QTL_PE_5, from 848,271,255 bp to 854,871,255 bp), 3D (QTL_RI_*attM*_2, from 42,030,6962 bp to 426,906,962 bp), and 6B (QTL_RI_ *expR^+^ch* _4 from 78,096,472 bp to 84,696,472 bp and QTL_RI_ *expR^+^ch* _5 from 86,582,943 to 93,182,943 bp) (Table 3). Two out of the four kinases (PROTEIN KINASE FAMILY PROTEIN AND MITOGEN-ACTIVATED PROTEIN KINASE 1) under *expR^+^ch* at physical position (82,924,787) on chromosome 6B were closely located at 23,005 bp of “BS00067590_51”. This region might be important in enhancing plant defense against pathogens, according to previous results (Schikora et al. [69]; Shrestha et al. [18]). These studies revealed that activated mitogen-activated protein kinases increased pathogen resistance in Arabidopsis and barley.

In addition to the mentioned genes, two important genes, namely cellulose synthase and callose synthase, were identified at QTL_RI_ *expR^+^ch* _3 and QTL_RI_ *expR^+^ch* _7 on chromosomes 6A and 6B, respectively. The synthesis of cellulose and callose may be involved in protection from different environmental stressors [70]. Plants respond to abiotic and biotic factors with local callose accumulation, which leads to a build-up of papillae at the sites of infection and induced resistance against plant pathogens [70,71,72]. Cellulose, like callose, plays a pivotal role in growth and defense in plants. Cano-Delgado et al. [73] pointed to reduced cellulose synthesis, which leads to lignification and defense responses by jasmonate and ethylene, as well as other signaling pathways in Arabidopsis plants. Menna et al. [74] reported enhanced resistance to three leaf-biotrophic pathogens by activating jasmonate and ethylene signaling pathways in cellulose-deficient mutants.

To the best of our knowledge, the presented study is the first report on QTL regions and associated markers for bacterial AHL-priming and bacterial AHL-priming efficiency for response to leaf rust in wheat. While several studies were conducted to identify QTLs associated with leaf rust via GWASs, no QTL has yet been reported for bacterial AHL priming in wheat. The identified QTL regions were compared with QTL regions from the literature [6,25,75,76] that used the genotyping platform (90K and 15K). The comparison indicated that marker “RAC875_c56205_127” identified for RI under *expR^+^ch* on chromosome 2A was located at 154,283 bp of the markers identified by Fatima et al. [75]. In addition, the marker “CAP8_c359_95” identified for PE at the physical position 74,742,550 on chromosome 3A was located at 287,872 bp of markers for infection type identified by Fatima et al. [75]. Two previous studies used 35K Axiom data for GWASs [3,24]. No overlapping marker could be identified by comparing our findings with these studies. The development of KASP (Kompetitive Allele-Specific PCR) markers based on the important markers identified by GWASs in our study could provide a useful tool to introduce resistance and bacterial AHL priming-related loci in elite breeding lines.

## 5. Conclusions

This study is the first report on the application of bacterial AHL priming to improve leaf-rust resistance in wheat. We showed genotype-specific responses to bacterial AHL priming and identified QTL regions, as well as putative candidate genes, associated with bacterial AHL-priming efficiency and relative infection. Twenty genotypes showed a significant positive effect on bacterial AHL-priming efficiency when treated with the *expR^+^ch* bacterial strain. Using GAPIT for GWAS, 24 significantly associated markers for RI under *attM* or *expR^+^ch* conditions and PE were identified. The information about primable genotypes, the identified QTL regions, and putative candidate genes represents a desirable genetic resource for improving wheat breeding for leaf-rust resistance. The introduction of AHL-primable genotypes could be an effective way to cope with the negative impact of biotic and abiotic factors in wheat production.

## Figures and Tables

**Table 1:** Descriptive statistics for the relative infection (RI) under *attM* and *expR^+^ch* and priming efficiency (PE).

Trait	Priming	Mean	Min	Max	SD	CV (%)	Repeatability
*Puccinia triticina* (scores)	*attM*	1.97	0	3.75	0.87	44.19	0.96
	*expR^+^ch*	1.89	0	3.75	0.83	43.91	0.97
*Puccinia triticina* (%)	*attM*	20.02	0	80.00	17.49	87.34	0.82
	*expR^+^ch*	16.42	0	80.00	14.10	85.90	0.84
Relative infection (RI)	*attM*	2.45	0.5	4.35	1.11	45.40	0.97
Relative infection (RI)	*expR^+^ch*	2.33	0.5	4.21	1.07	45.87	0.98
Priming efficiency (PE)		0.11	−2.85	2.68	0.46	414.95	0.82

**Table 2:** Analysis of variance (ANOVA) for relative infection (RI).

Effect	F Value	*p*-Value
Genotype	355.9	<0.001
Priming effects	178.5	<0.001
Genotype × priming effects	1.9	<0.001

**Table 3:** List of significant (−log_10_ (*p*-value) > 3) markers that were uniquely identified for PE, RI under *attM*, and RI under *expR^+^ch*.

Priming	Trait	QTL	SNP	Chr ^a^	Pos ^b^	*p*-Value	−log_10_P ^c^	R^2 d^	SNP Effect	Annotation
*attM*	RI	QTL_RI_*attM*_1	RAC875_c11652_544	2A	34,649,538	<0.001	3.14	0.30	0.23	Eukaryotic aspartyl protease family protein
	RI		BobWhite_c2022_245	2A	34,661,266	<0.001	3.14	0.30	0.23	Glucose-6-phosphate 1-dehydrogenase
	RI	QTL_RI_*attM*_2	GENE-1805_65	3D	423,606,962	<0.001	3.04	0.30	−0.20	Kinase family protein
	RI	QTL_RI_*attM*_3	RAC875_c56205_127	6B	721,632,069	<0.001	3.19	0.31	0.30	ATP-dependent RNA helicase
*expR^+^ch*	RI	QTL_RI_ *expR^+^ch* _1	Tdurum_contig43943_56	1A	11,438,538	<0.001	3.25	0.29	−0.49	Chaperone protein DnaJ
	RI	QTL_RI_ *expR^+^ch* _2	BS00068817_51	3B	622,821,075	<0.001	3.07	0.29	0.34	LexA repressor
	RI	QTL_RI_ *expR^+^ch* _3	GENE-0221_350	6A	50,835,408	<0.001	3.20	0.29	−0.28	Cellulose synthase
	RI	QTL_RI_ *expR^+^ch* _4	RAC875_c10650_90	6B	80,018,996	<0.001	3.23	0.29	0.28	Aminoalcoholphosphotransferase 1
	RI	QTL_RI_ *expR^+^ch* _4	Kukri_c32307_481	6B	81,396,472	<0.001	3.64	0.30	−0.30	Cytochrome P450
	RI		TA005332-1378	6B	82,034,544	<0.001	3.23	0.29	0.28	50S ribosomal protein L4
	RI		BS00067590_51	6B	82,924,787	<0.001	3.23	0.29	−0.28	Protein kinase family protein and Mitogen-activated protein kinase 1
	RI		Kukri_c17622_298	6B	82,951,636	<0.001	3.42	0.29	−0.29	NBS-LRR-like resistance protein
	RI	QTL_RI_ *expR^+^ch* _5	Excalibur_s111479_146	6B	89,882,943	<0.001	3.20	0.29	0.28	Protein kinase
	RI	QTL_RI_ *expR^+^ch* _6	RAC875_c14309_317	6B	659,313,464	<0.001	3.02	0.29	0.26	Lysine ketoglutarate reductase/saccharopine dehydrogenase
	RI	QTL_RI_ *expR^+^ch* _7	Kukri_c11397_2523	6B	729,834,583	<0.001	3.26	0.29	−0.27	Callose synthase
	PE	QTL_PE_1	wsnp_Ex_c39616_46871127	1B	569,054,340	<0.001	3.37	0.05	−0.05	H/ACA ribonucleoprotein complex non-core subunit NAF1
	PE		BS00021710_51	1B	569,563,462	<0.001	3.89	0.07	0.06	Secretory carrier-associated membrane protein
	PE	QTL_PE_2	CAP8_c359_95	3A	74,742,550	<0.001	3.22	0.05	0.06	Clavaminate synthase-like protein
	PE	QTL_PE_3	wsnp_Ex_c1538_2937905	3A	219,753,903	< 0.001	4.14	0.08	−0.10	Peroxidase
	PE	QTL_PE_4	Tdurum_contig31586_197	3A	512,355,527	<0.001	3.02	0.04	0.05	RING/U-box superfamily protein
	PE	QTL_PE_5	Tdurum_contig59566_1534	3B	851,570,483	<0.001	3.19	0.05	0.05	Serine/threonine-protein kinase ATM
	PE		Kukri_c55981_194	3B	851,570,902	<0.001	3.32	0.05	−0.05	Serine/threonine-protein kinase ATM
	PE		Tdurum_contig59566_2309	3B	851,571,255	<0.001	3.44	0.06	0.05	Serine/threonine-protein kinase ATM
	PE		wsnp_JD_c18509_16968425	3B	851,572,724	<0.001	3.24	0.05	0.05	Serine/threonine-protein kinase ATM

^a^ Chr = chromosome; ^b^ Pos = position of marker; ^c^ −log10P = −log_10_ of the *p*-value; ^d^ R^2^, variance explained by marker in %.

## Data Availability

The original contributions presented in the study are included in the article/Supplementary Materials, further inquiries can be directed to the corresponding author.

## Supplementary Materials

The following supporting information can be downloaded at: https://www.mdpi.com/article/10.3390/microorganisms12101936/s1, Table S1. List of 175 genotypes used in this study with information about origin, genotyping platform, and assigned priming efficiency (PE). Table S2. Pearson correlation coefficients between experiments for *Puccinia triticina* (Scores), *Puccinia triticina* (scores), and relative infection (RI). Table S3. Data for relative infection (RI) and priming efficiency (PE) for the 175 genotypes across the three experiments. Table S4. List of calculated LD decay for each chromosome, separately. Table S5. List of all identified significant (LOD > 3) markers for relative infection under *attM* and *expR^+^ch* treatment. The 13 common markers observed between *attM* and *expR^+^ch* for RI are highlighted in red. Table S6. List of identified candidate genes located within the identified QTL regions and GO terms. Table S7. Information on GO terms for identified genes in QTL regions. Figure S1. Priming experiment with *attM* (left side) and *expR^+^ch* (right side) in the greenhouse. Figure S2. Boxplot based on genotype means for (a) *Puccinia triticina* (scores), (b) *Puccinia triticina* (%), and (c) relative infection (RI). Figure S3. Number of optimal detected clusters (K = 2) according to Bayesian clustering approach. Figure S4. Q-Q plot for priming efficiency and each tested method to conduct GWASs separately.
