# Supplementary figures and images for: Bacterial N-Acyl Homoserine Lactone Priming Enhances Leaf-Rust Resistance in Winter Wheat and Some Genomic Regions Are Associated with Priming Efficiency
Source: Microorganisms. 2024 Sep 24;12(10):1936. doi: 10.3390/microorganisms12101936 (PMC11509450; doi:10.3390/microorganisms12101936)

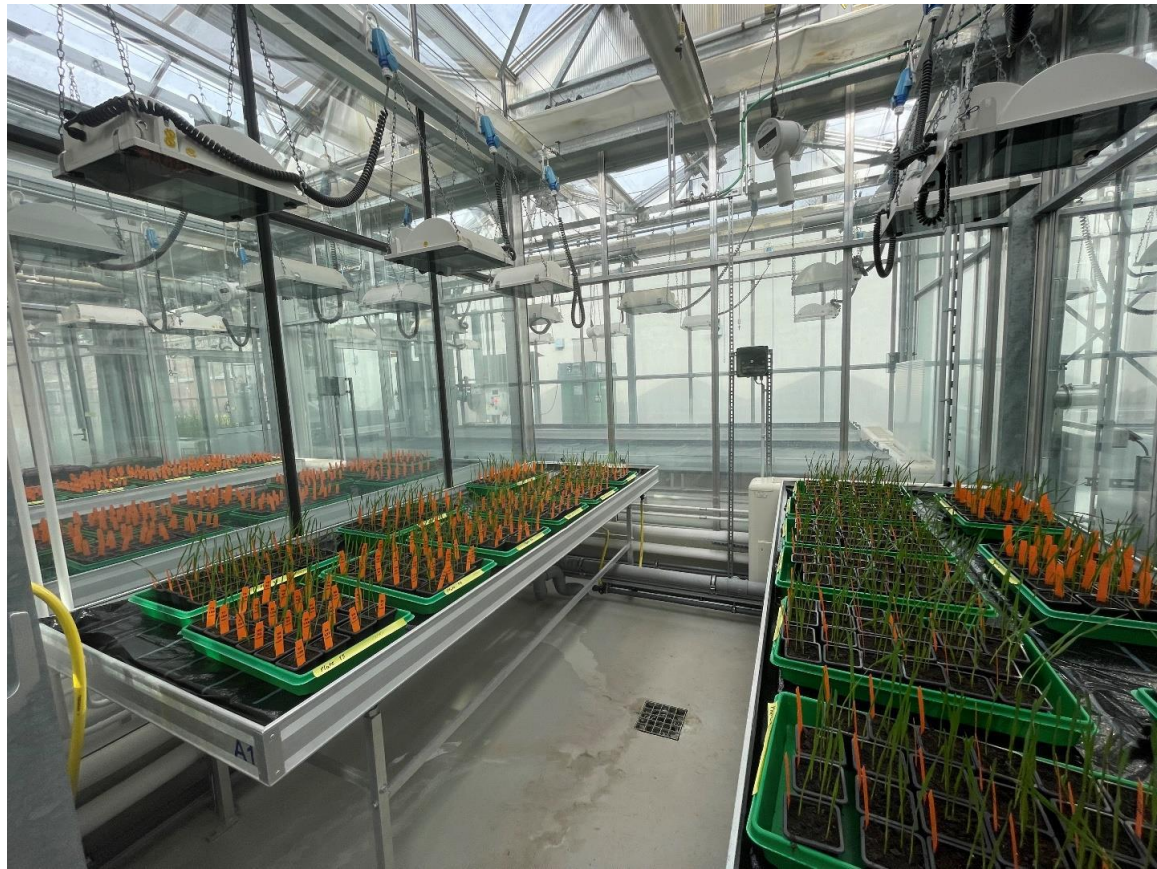

Supplement: Supplementary file 1 [file microorganisms-12-01936-s001.zip › Figure S1.pdf]

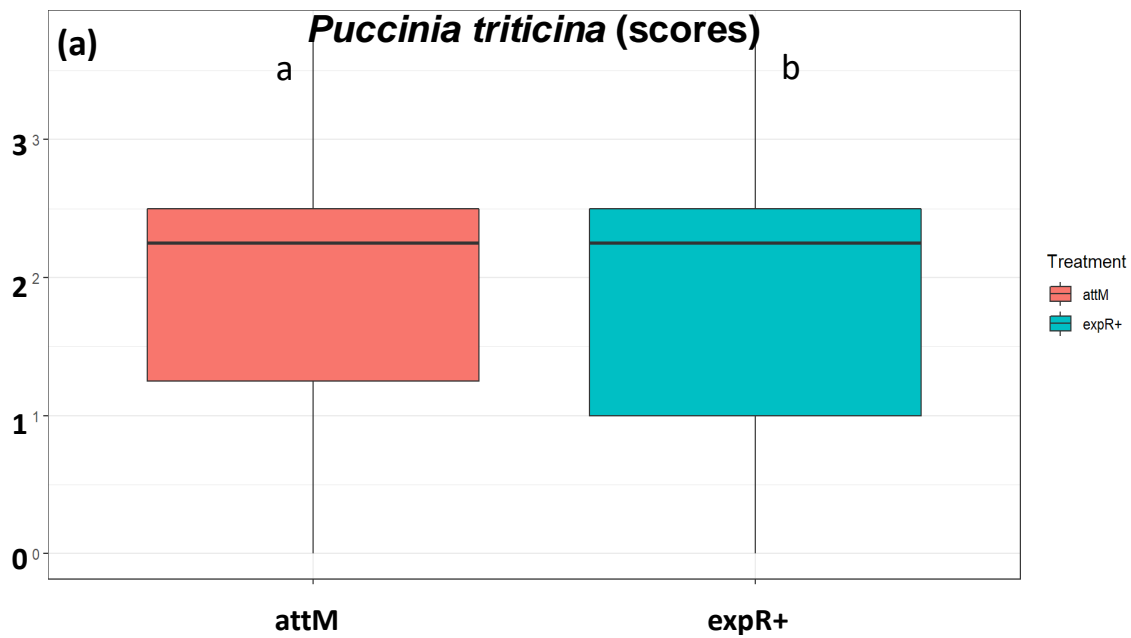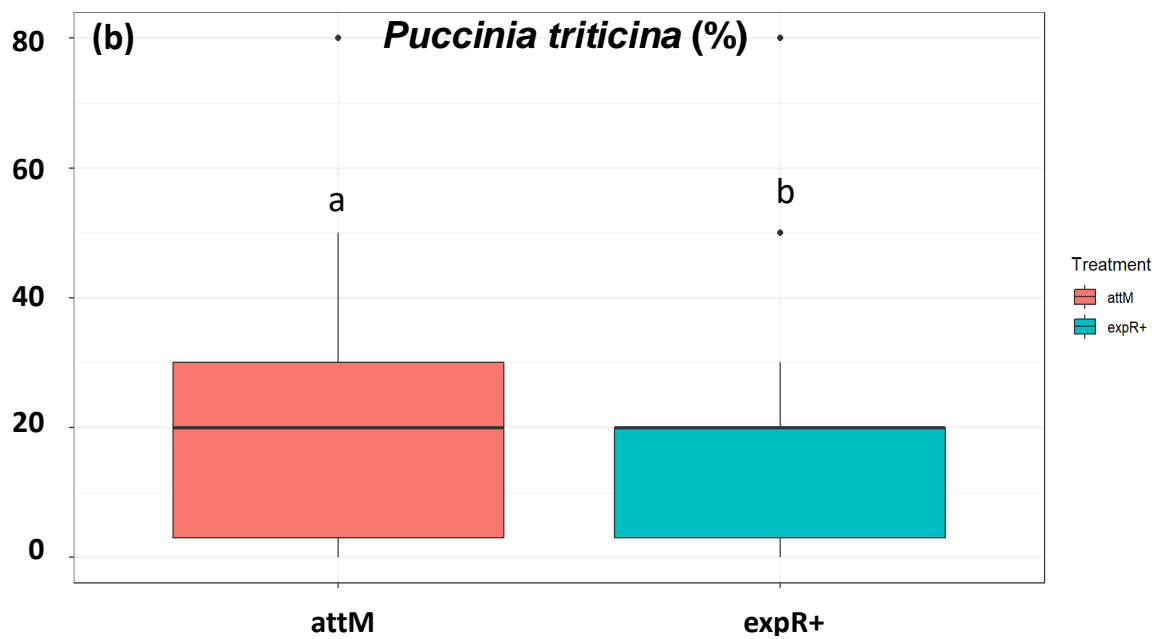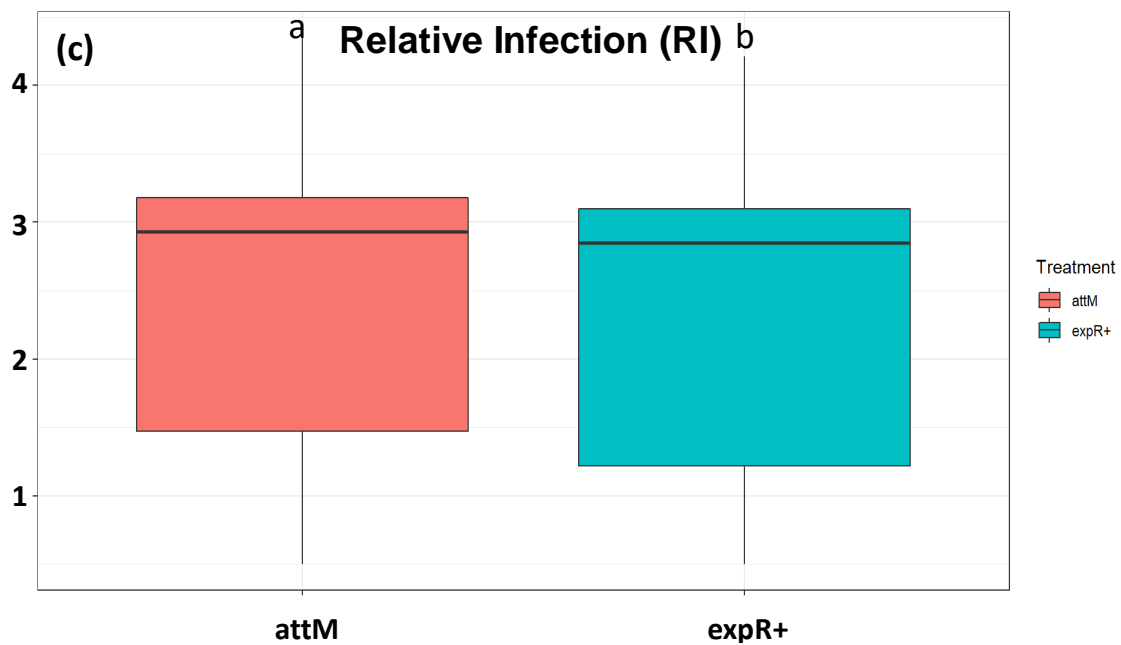

Supplement: Supplementary file 1 [file microorganisms-12-01936-s001.zip › Figure S2.pdf]

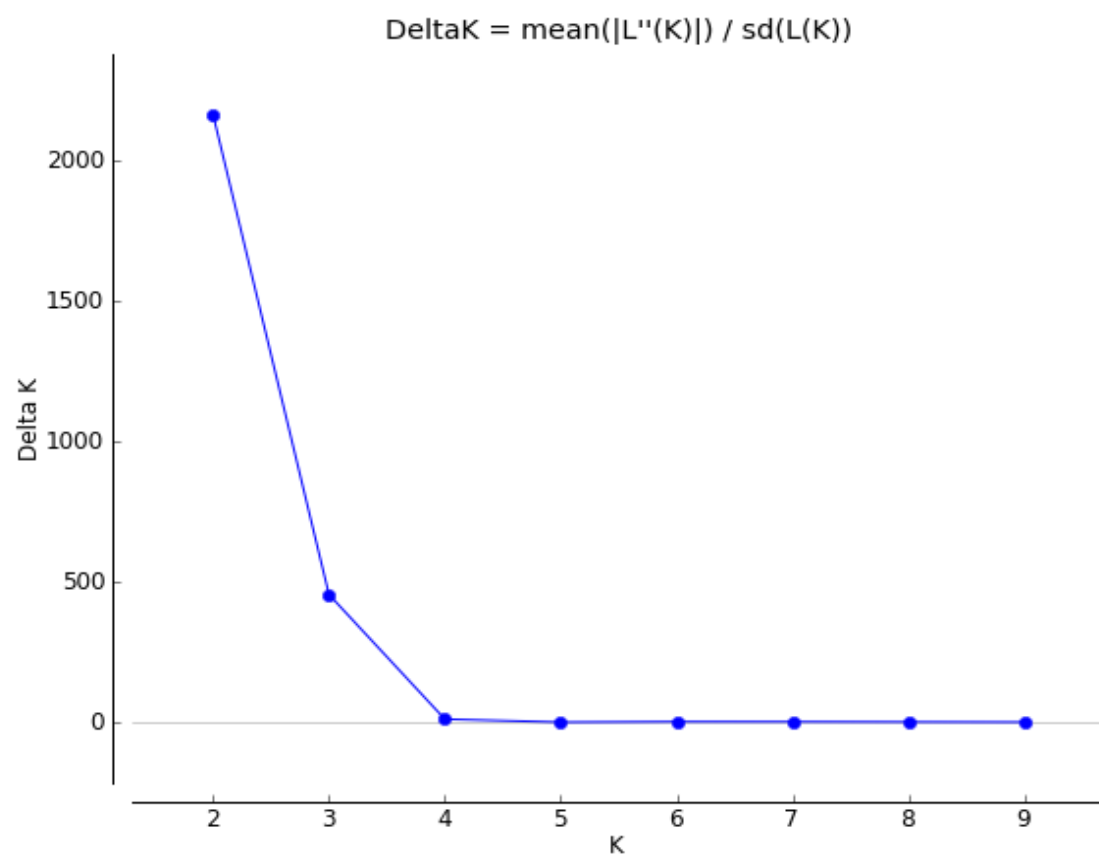

Supplement: Supplementary file 1 [file microorganisms-12-01936-s001.zip › Figure S3.pdf]

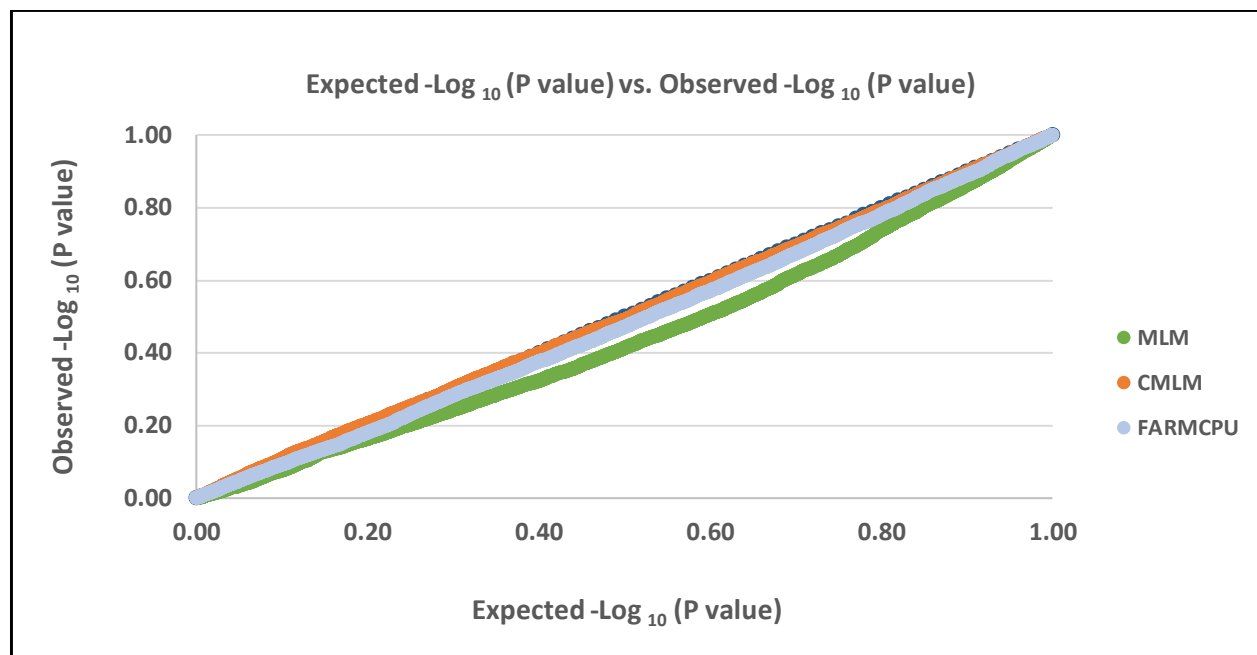

Supplement: Supplementary file 1 [file microorganisms-12-01936-s001.zip › Figure S4.pdf]
